# Physical and electrical properties of Cu_2_CoSnS_4_ nanoparticles synthesized by hydrothermal growth at different reaction time and copper concentration

**DOI:** 10.1016/j.dib.2020.106103

**Published:** 2020-07-30

**Authors:** Mokurala Krishnaiah, Ajit Kumar, Sung Hun Jin, Junyoung Song

**Affiliations:** Department of Electronic Engineering, Incheon National University, Incheon 406-772, Republic of Korea

**Keywords:** Hydrothermal process, hydrothermal reaction time, chemical composition, XPS analysis and TLM patterns I-V characteristics

## Abstract

Herein, the physical properties such as crystal phase, morphology, and chemical composition for Cu_2_CoSnS_4_ (CCTS) nanoparticles, synthesized by hydrothermal growth at 200 °C, are studied according to the short reaction times from 1 h to 6 h, respectively. The raw data of chemical composition, XPS analysis, and their electrical properties of CCTS nanoparticles prepared at 200 °C for 12 h with different Cu concentrations are presented [1] in the present manuscript. Materials properties of CCTS and their electrical, optical properties were systematically studied, and their correlation between physical properties and materials properties is strongly studied in depth mode.

Specifications table

**Table:** 

Subject	Physics, Electronic Engineering
Specific subject area	Materials Science, Photodetector
Type of data	Table Figure
How data were acquired	X-ray diffraction (XRD, Rigaku, smart Lab, the 2-theta scan range of 20ᵒ–80ᵒ using Cu _Kα_ radiation (λ = 1.54 Å) irradiation) Field emission scanning electron microscope (FESEM, JOEL, JSM_7800 F, operating voltage =15kV and working distance (10 mm)) Energy dispersive spectroscopy (EDS, JEOL, EDS-7800F) measurements of the synthesized nanoparticle were performed to quantify the chemical composition (operating voltage =15 kV and working distance (10mm)) X-ray photoelectron spectroscopy (XPS, PHI 5000 VersaProbe ll) The absorbance properties of prepared films were m easured using UV–Vis–NIR Spectrometer (Agilent Technologies) in the wavelength ranging from 400 nm to 1100 nm I-V measurements for two-terminal devices on CCTS films were carried out by usin a semiconductor parameter analyzer (Agilent, 4155B).
Data format	Raw and analyzed
Parameters for data collection	X-ray diffraction (XRD, Rigaku, smart Lab, the 2-theta scan range of 20ᵒ–80ᵒ using Cu _Kα_ radiation (λ = 1.54 Å) irradiation) Field emission scanning electron microscope (FESEM, JOEL, JSM_7800 F, operating voltage =15 kV and working distance 10mm) Energy dispersive spectroscopy (EDS, JEOL, EDS-7800F) measurements of the synthesized nanoparticle were performed to quantify the chemical composition (operating voltage =15 kV and working distance (10 mm)) X-ray photoelectron spectroscopy (XPS, PHI 5000 VersaProbe ll, operating voltage:3 kV, full scan time 1 h) The absorbance properties of prepared films were measured using UV–Vis–NIR Spectrometer (Agilent Technologies) in the wavelength ranging from 400 nm to 1100 nm I-V measurements for two-terminal devices on CCTS films were carried out by using a semiconductor parameter analyzer (Agilent, 4155B) (−5 V to 5 V)
Description of data collection	Growth of CCTS nanoparticles via a hydrothermal process
Data source location	Incheon National University, Incheon 22012, Korea
Data accessibility	The data are with this article
Related research article	Mokurala Krishnaiah, Ajit Kumar, Song Jun-Young*, and Jin Sung Hun* “Cu/(Co+Sn) ratio effects on physical and photodetective properties for visible light absorbing Cu_2_CoSnS_4_ nanoparticles via a one-pot hydrothermal process.” Journal of alloys and compounds for a co-submission research article in press (10.1016/j.jallcom.2020.156174)

Value of the Data

• The study of chemical composition and morphology of CCTS nanoparticles synthesized at different hydrothermal reaction times can be useful to guide design rules for obtaining an optimal chemical composition of the light absorber for the better thin-film solar cell performance
• XPS data are useful to identify the surface contaminates and thin oxide layer formation on the synthesized CCTS nanoparticles
• The calculated optical absorption coefficients values are beneficial to the design of the light-absorbing layer for thin-film solar cells and other optoelectronic devices
• TLM pattern-based current-voltage characteristics of CCTS nanoparticles, synthesized with different Cu concentrations, is a simple technique to calculate the resistivity, contact resistance, and sheet resistance of two-terminal devices.

## Data description

**Fig. 1:**
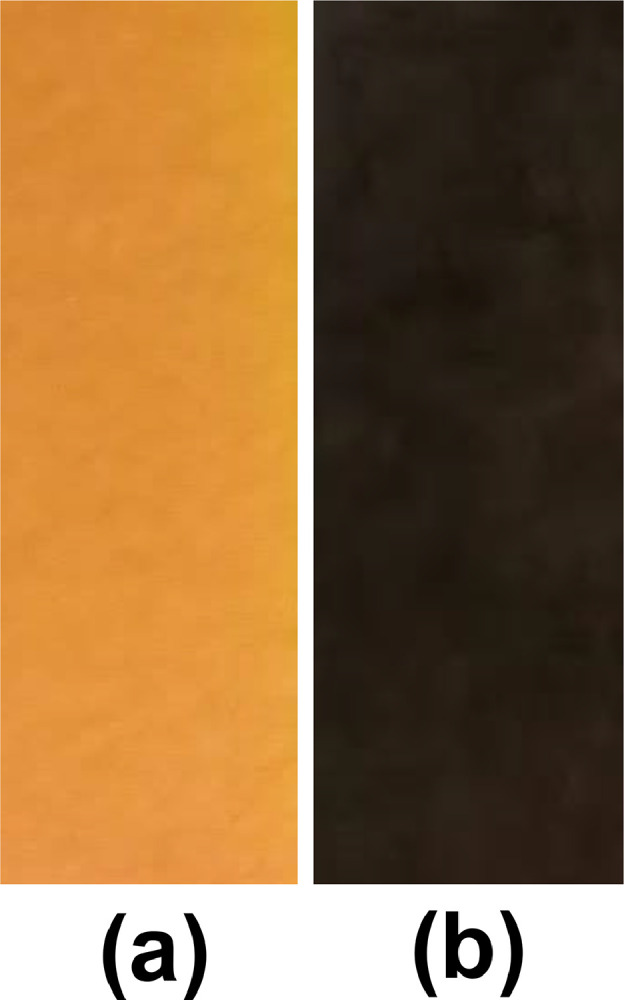
PH level change of CCTS precursor's solution (a) before and (b) after addition of 7 mlMEA.

The process parameters such as usage of initial precursors (and their concentration), surfactant /complexing agent, pH value, and reaction temperature/time are controlling a phase purity, chemical composition, and morphology over synthesized nanoparticles by hydrothermal process [1], [2], [3], [4], [5], [6], [7], [8], [9], [10]. The change in pH value of CCTS precursor's solution before and after the addition of MEA is shown in Fig. 1. The CCTS precursor's solution changes from acid to base nature after the addition of MEA.

**Fig. 2:**
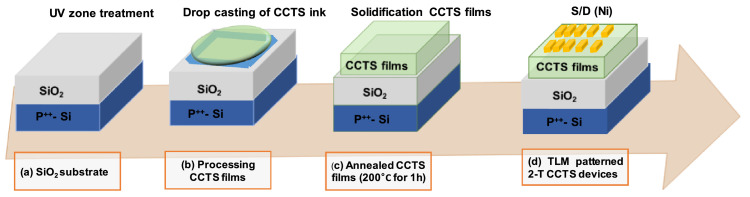
Sequence of the fabrication process for two-terminal CCTS devices on the heavily boron-doped Si substrates with thermal oxide.

All synthesized CCTS nanoparticles were further utilized to study the effects of Cu concentrations on electrical and photoresponse of CCTS nanoparticles-based films. 200 mg synthesized CCTS nanoparticles dispersed in 1 ml N-Methyl-2-pyrrolidone (NMP) in a vial and ultrasonicated for 15 min to form a homogeneous paste. The prepared paste was coated on thermally oxidized Si wafer (300 nm) by drop-casting technique to form a film. The drop casted films were annealed in vacuum at 200 °C for 1 h. Two terminal CCTS devices were fabricated by depositing Ni as a drain and source via thermal evaporation. The detailed two-terminal device fabrication steps are shown in Fig. 2. The electrical properties, such as sheet resistance of fabricated devices, were measured via the transfer line method (TLM).

**Fig. 3:**
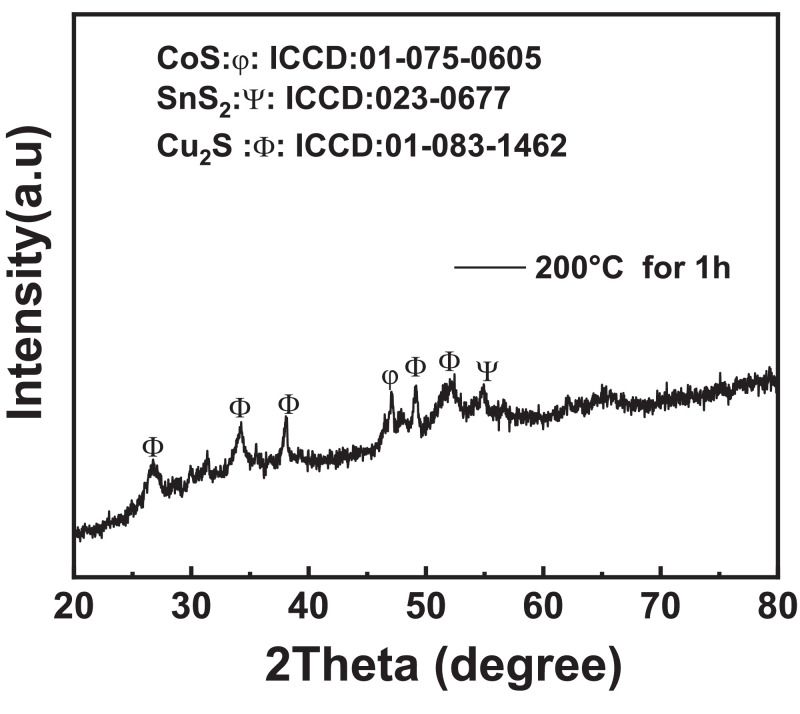
XRD pattern of CCTS nanoparticles synthesized by the hydrothermal process at 200 °C for 1 h.

The formation of the secondary phases for CCTS nanoparticles synthesized at 200 °C for 1 h, is verified by the analysis of the XRD pattern, as shown in Fig. 3. The result indicates that 1 h growth time for CCTS nanoparticles is not sufficient for the complete reaction and its growth of CCTS nanoparticles.

**Fig. 4:**
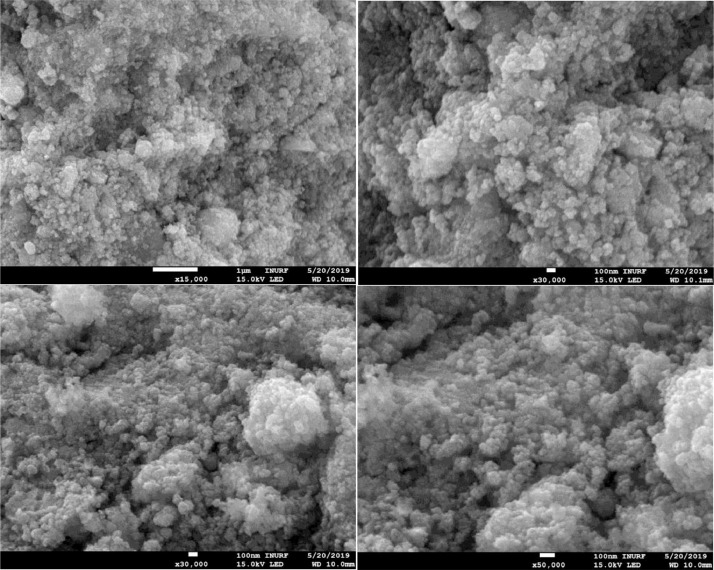
FEG-SEM images for CCTS nanoparticles synthesized with 1.90 mmol Cu via MEA promoted hydrothermal process at 200 °C for 3 h

**Fig. 5:**
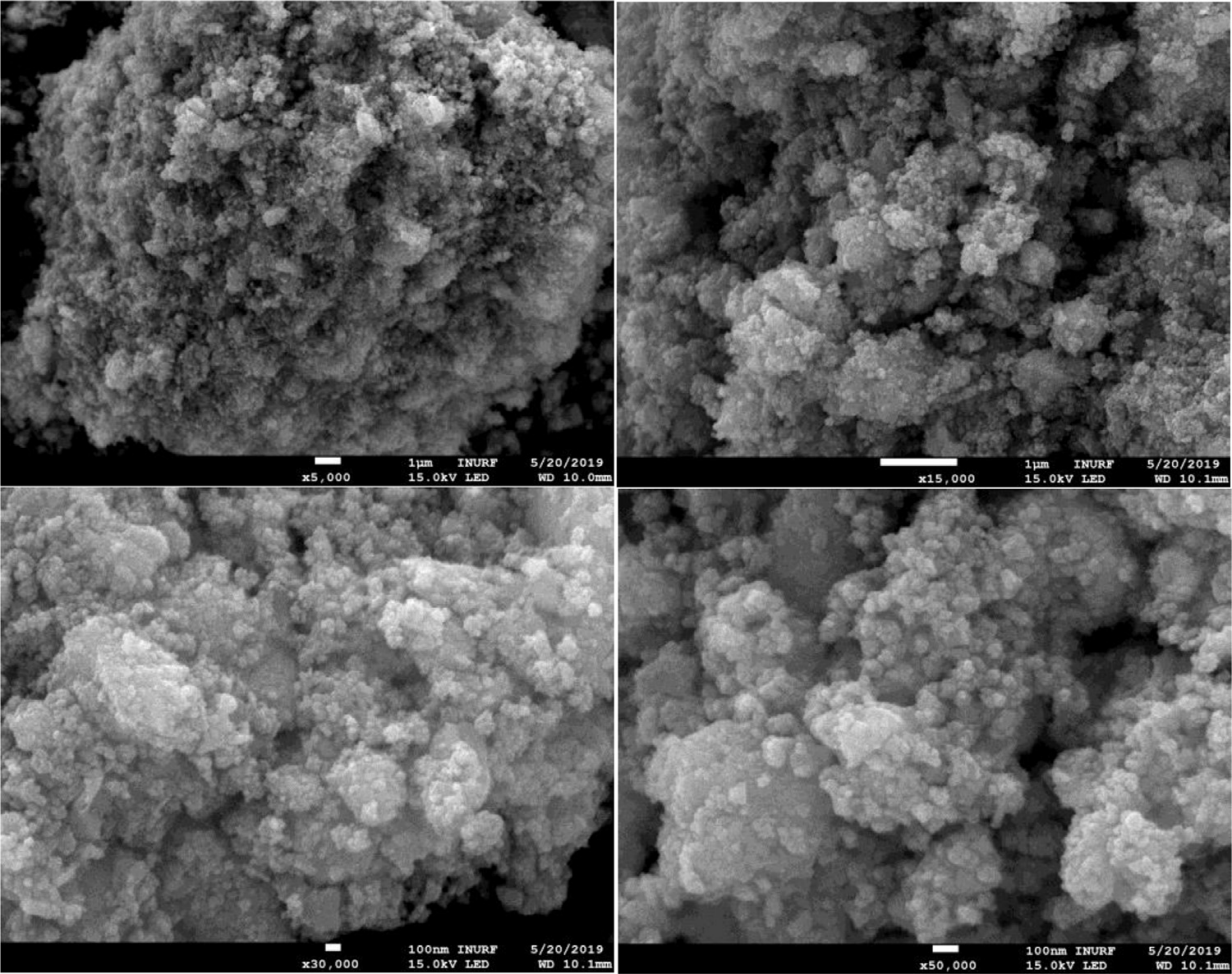
FEG-SEM images of CCTS nanoparticles synthesized with 1.90 mmol Cu via MEA promoted hydrothermal process at 200 °C for 6 h

**Fig. 6:**
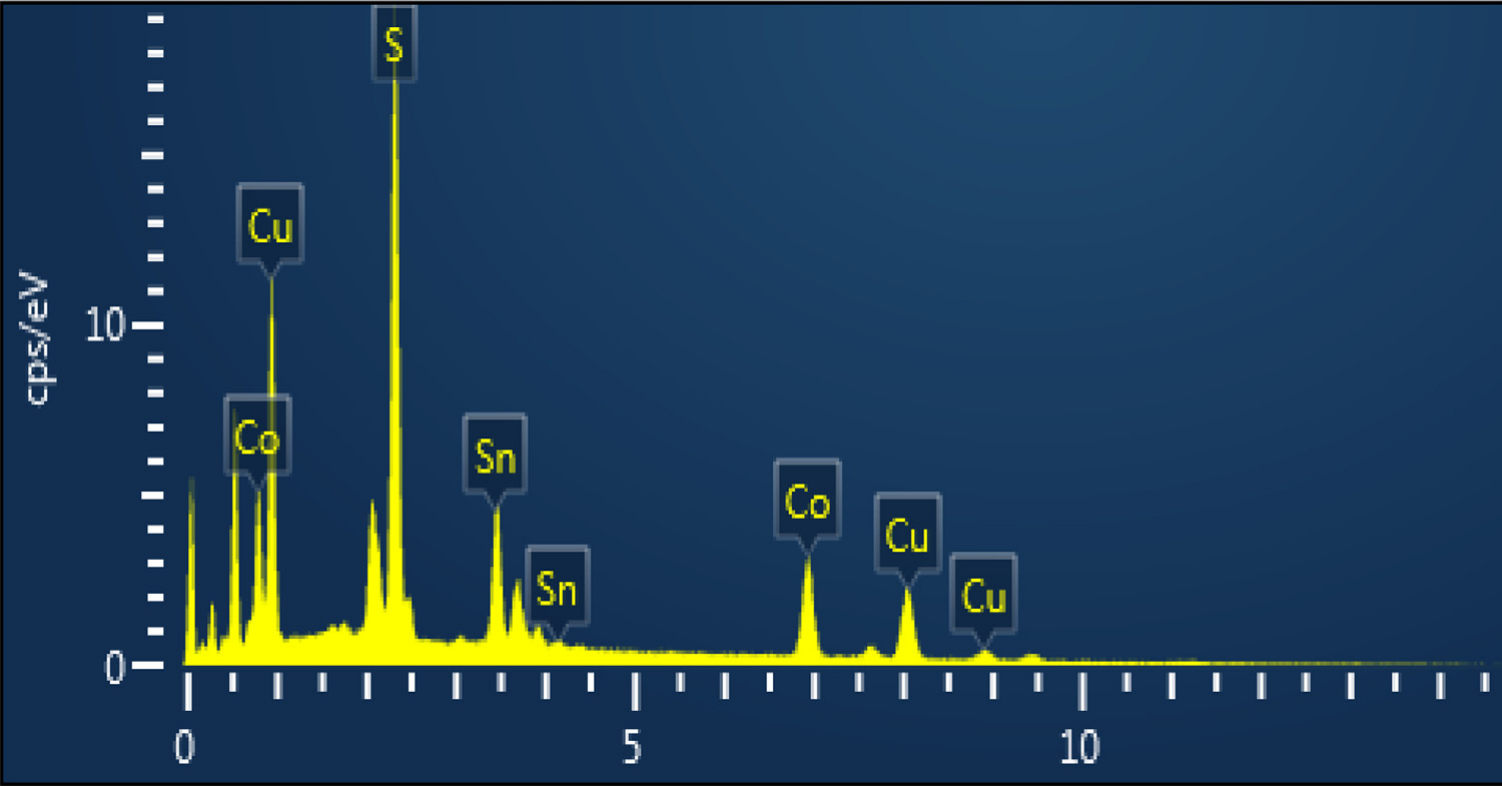
EDS spectra for CCTS nanoparticles synthesized with 1.90 mmolCu via MEA promoted hydrothermal process at 200 °C for 1 h

**Fig. 7:**
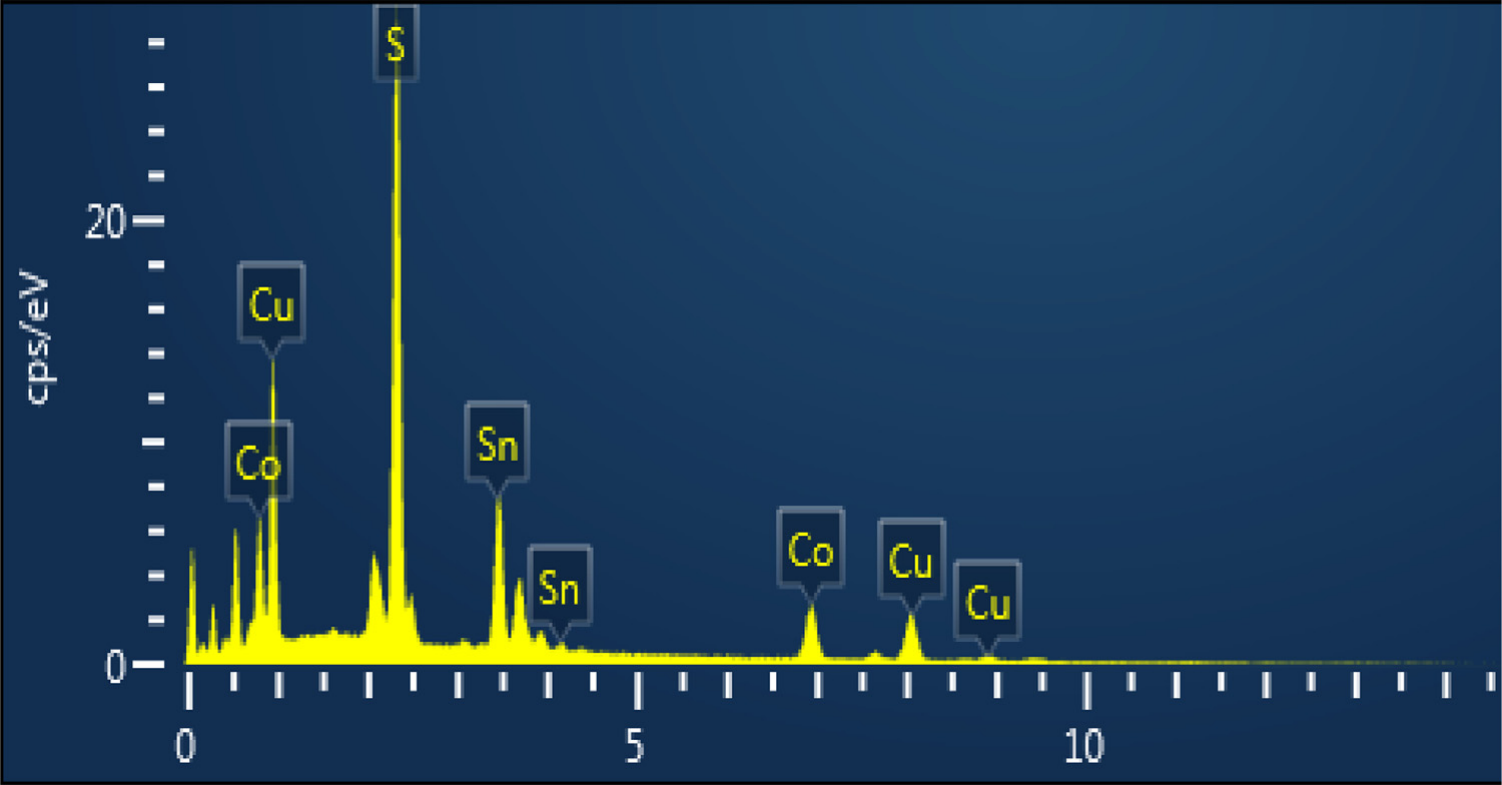
EDS spectrum for CCTS nanoparticles synthesized with 1.90 mmol Cu via MEA promoted hydrothermal process at 200 °C for 3 h

**Fig. 8:**
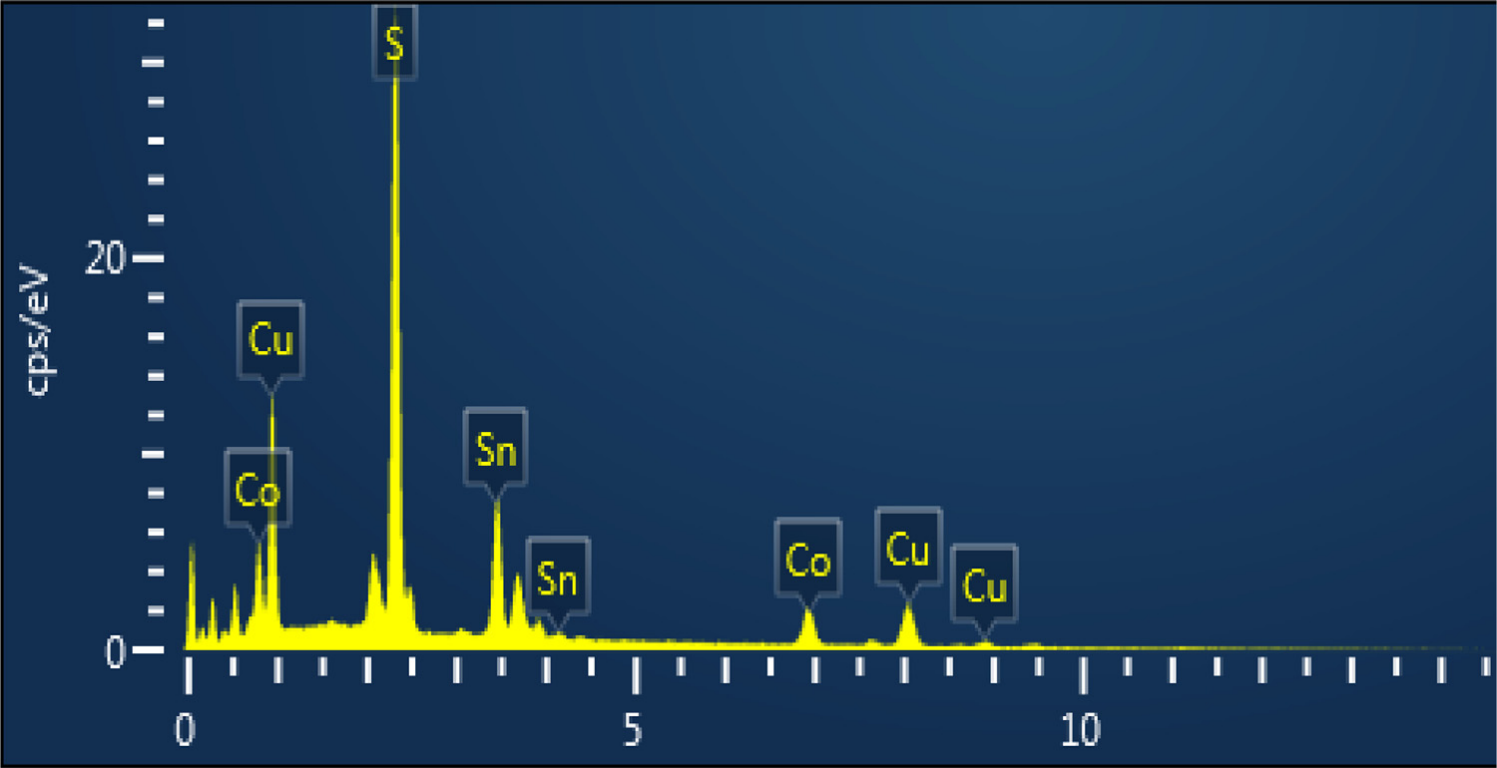
EDS spectra for CCTS nanoparticles synthesized with 1.90 mmolCu via MEA promoted hydrothermal process at 200 °C for 6 h

The morphology of CCTS nanoparticles, synthesized at 200 °C for 3 h and 6 h, respectively, are shown in Fig. 4, Fig. 5. The morphology of synthesized nanoparticles looks like a spherical shape. The EDS spectrum of CCTS nanoparticles, synthesized for 1 h, 3 h, and 6 h, respectively, are shown in Fig. 6, Fig. 7, Fig. 8. The raw data are listed in Tables 3 and 4. The Cu-rich, Co – rich and Sn and S-deficient are noticed for short reaction times (1 h–3 h), and stoichiometric chemical composition is observed for nanoparticles synthesized for 6 h. Similar trends were seen in CZTS nanoparticles synthesized by a hydrothermal process [[1], [2],^6^].

**Fig. 9:**
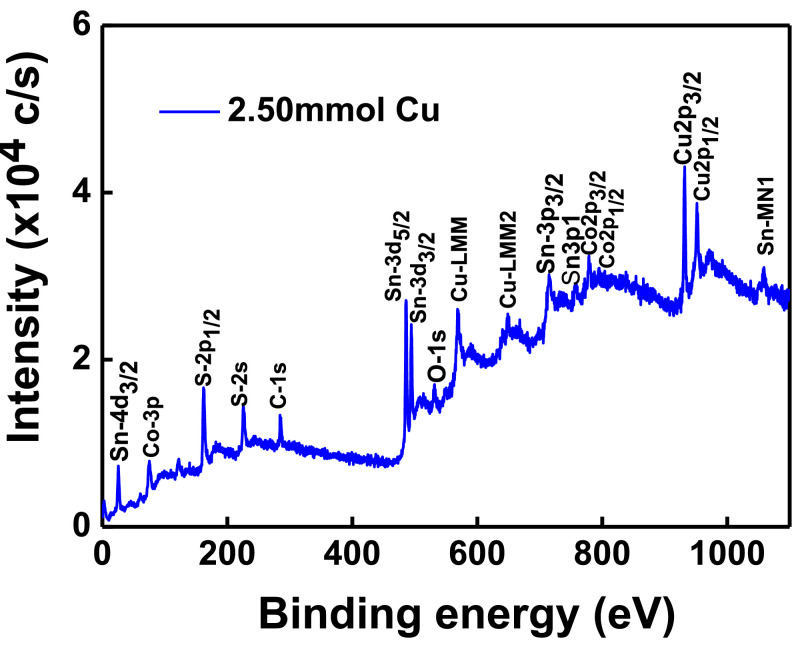
XPS scan survey spectrum for CCTS nanoparticles synthesized with 2.50 mmol Cu via MEA promoted hydrothermal process at 200 °C for 12 h.

**Fig. 10:**
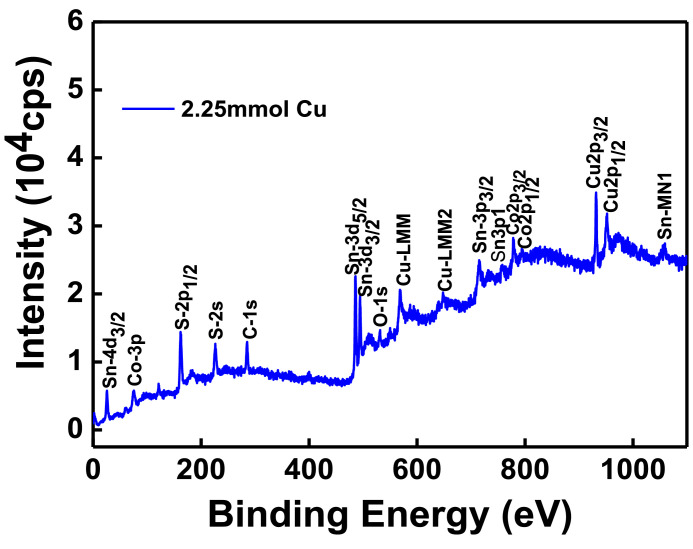
XPS scan survey spectrum for CCTS nanoparticles synthesized with 2.25 mmol Cu via MEA promoted hydrothermal process at 200 °C for 12 h

**Fig. 11:**
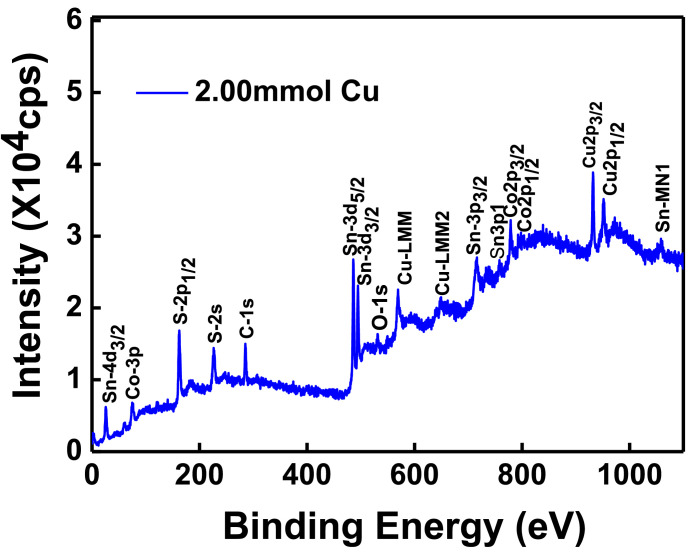
XPS scan survey spectrum for CCTS nanoparticles synthesized with 2.00 mmol Cu via MEA promoted hydrothermal process at 200 °C for 12 h

**Fig. 12:**
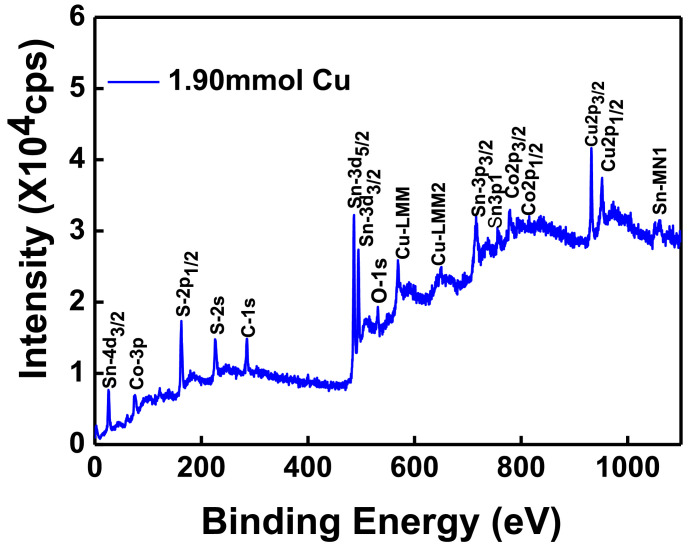
XPS scan survey spectrum for CCTS nanoparticles synthesized with 1.90mmol Cu via MEA promoted hydrothermal process at 200 °C for 12 h

**Fig. 13:**
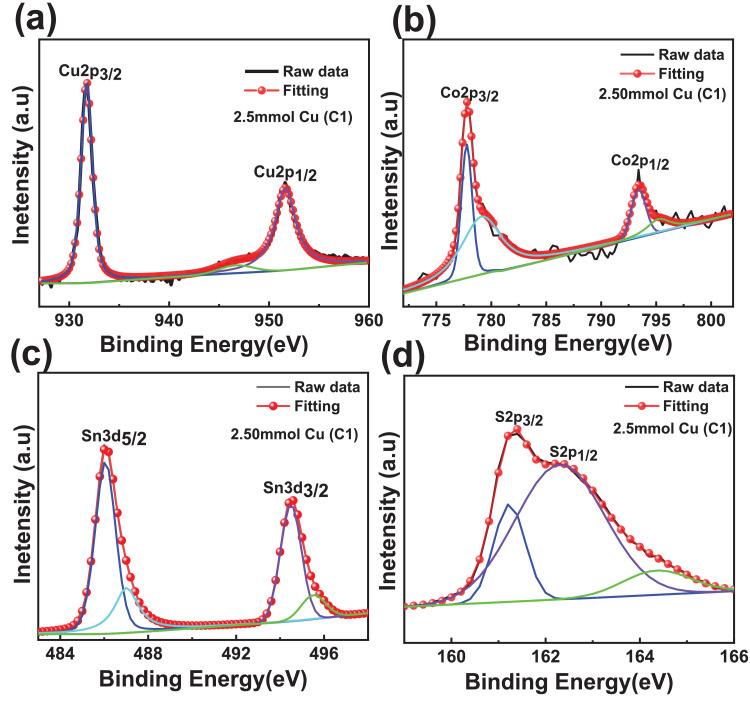
Fitted core levels of XPS spectra for constituent elements of synthesized CCTS nanoparticles with 2.50 mmol Cu (C1) respectively.

**Fig. 14:**
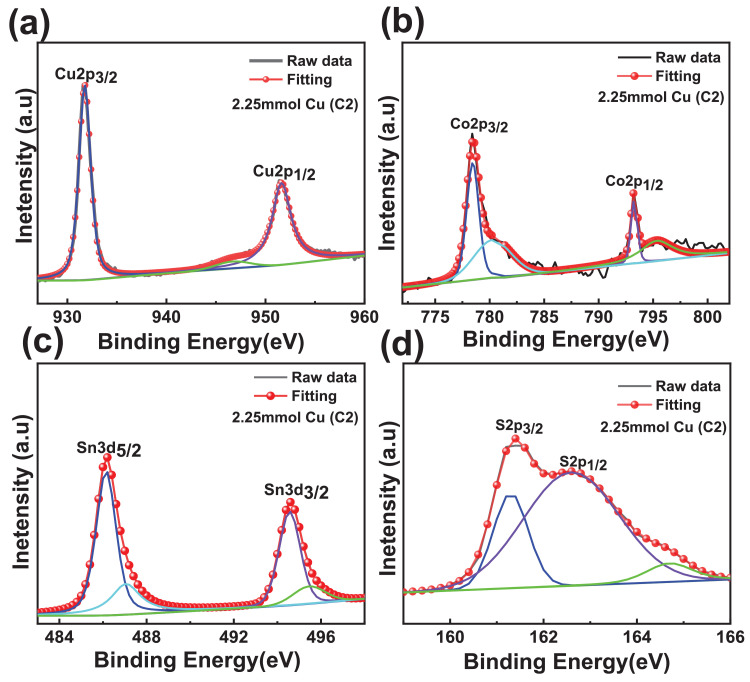
Fitted core levels XPS spectra of constituent elements of synthesized CCTS nanoparticles with 2.25 mmol Cu (C2) respectively.

**Fig. 15:**
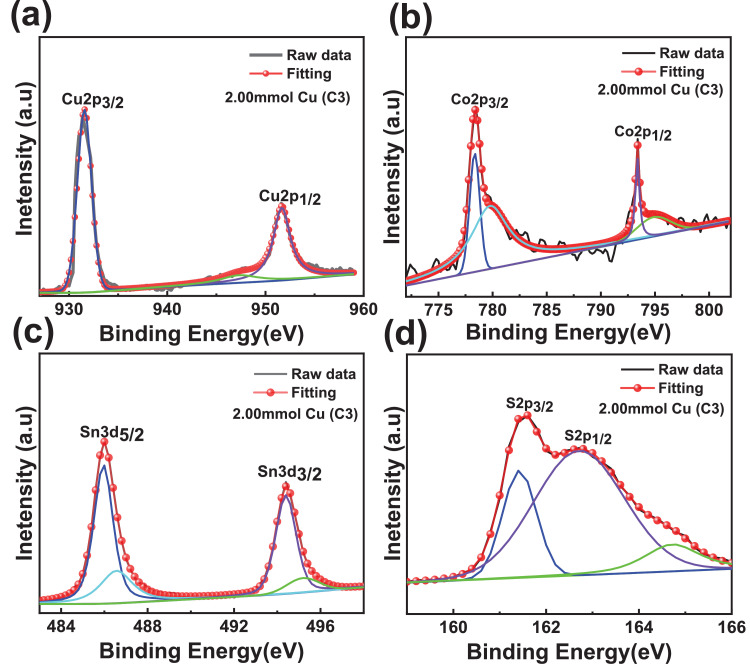
Fitted core levels XPS spectra of constituent elements of synthesized CCTS nanoparticles with 2.00 mmol Cu (C3) respectively

The XPS scanning surveyed-spectra for CCTS nanoparticles synthesized with different concentrations of Cu precursors, corresponding to 2.50 mmol, 2.25 mmol, 2.00 mmol, and 1.90 mmol, respectively, are shown in Fig. 9–12. All consistent elements Cu, Co, Sn, and S of CCTS nanoparticles are detected in Fig. 9–12. The additional elements, like oxygen and carbon, are observed in the spectra. The carbon peak is attributed to the reference sample, and oxygen is possibly due to the contact in the air during the sample preparation and deionized water (DW) as a solvent. The elemental composition of CCTS nanoparticles, acquired from the XPS analysis, is in accordance with EDS analysis, and the raw data are listed in Tables 1, 2, and 5. The high-resolution spectra of constituent elements are fitted with XPS PEAK 41 software (Fig. 13–15). The peaks positions and FWHM values of synthesized nanoparticles are summarized in Table 6–9. The fitted core-level spectra of Cu2p shows two peaks Cu2p_3/2_ (931.74 eV–931.575 eV) and Cu2p_1/2_ (951.624 eV–951.525 eV) respectively (Figs. 13a–a). The split difference is found to be 19.84 eV. The calculated split difference values are similar to the reported for Cu^1+^ [6]. The satellite peak at 946.660 eV–947.317 eV is observed in all synthesized CCTS nanoparticles. The fitted core-level spectra of Co2p have displayed two splits Co2p_3/2_ (778.770 eV to778.350 eV), Co2p_1/2_ (793.408 eV to 793.302 eV), and the difference between these peaks are estimated to be 14.81 eV (Fig. 13, Fig. 14, Fig. 15). The splits separation agrees well with the reported value of Co^2+^ [6]. The extra peaks at 779.113 eV to795.220 eV and 795.220 eV to 795.220 eV are detected in the spectra (Fig. 8b), which is attributed to oxidation of Co (Co-O bonding) on CCTS nanoparticles surface during ambient exposure [11]. The core-level spectra of Sn3d represent two peaks, such as Sn3d_5/2_ (486.056 eV to 485.956 eV), Sn3d_3/2_ (494.491 eV–494.391 eV), and the separation between these peaks are determined to be 8.37 eV (Fig. 8c), indicating that Sn^4+^ is present in the CCTS films [6,11]. The other peaks at 486.561 eV–487.010 eV, 495.190 eV- 495.514 eV are noticed in spectra, thus demonstrating the presence of SnO_2_ on CCTS surface during the ambient exposure [9,11]. The fitted core level spectra of S are two peaks, such as S2p_1/2_, S2p_3/2_, with its peaks difference 1.20 eV (Fig. 2d), which is ascribed to S^−2^ in the CCTS films [9, 11]. The satellite peak is observed at 164 .70 eV, and thus, confirming that the SO_2_ is formed at the surface of CCTS particles [9].

**Table 1:** Chemical composition of CCTS nanoparticles synthesized (synthesized at 200 °C for 12 h) with different Cu concentrations is obtained from EDS analysis

Cu concentration	2.5 mmol	2.25 mmol	2.00 mmol	1.90 mmol
Cu	27.27 ± 1.18	25.56 ± 1.11	23.89 ± 1.32	22.30 ± 0.51
Co	14.37 ± 0.72	12.86 ± 0.94	13.29 ± 0.68	13.22 ± 1.24
Sn	8.92 ± 0.85	10.72 ± 1.08	11.99 ± 1.27	12.87 ± 1.22
S	49.43 ± 1.96	50.85 ± 1.31	50.81 ± 1.55	51.6 ± 0.37

**Table 2:** Compositional ratios of CCTS nanoparticles synthesized (synthesized at 200 °C for 12 h) with different Cu concentrations is obtained from EDS analysis

Cu concentration	2.5mmol	2.25mmol	2.00mmol	1.90 mmol
Cu/(Co+Sn)	1.17 ± 0.02	1.08 ± 0.04	0.94 ± 0.02	0.86 ± 0.06
Cu/Sn	3.07 ± 0.16	2.39 ± 0.13	2.00 ± 0.10	1.74 ± 0.12
Co/Sn	1.61 ± 0.07	1.20 ± 0.03	1.11 ± 0.06	1.02 ± 0.01
S/(Cu+Sn+Co)	0.97 ± 0.014	1.03 ± 0.04	1.03 ± 0.03	1.06 ± 0.06

**Table 3:** Chemical composition of CCTS nanoparticles synthesized with 1.90 mmol Cu at 200 °C for different reaction times is obtained from EDS analysis

Cu concentration	1 h	3 h	6 h	12 h
Cu	36.55 ± 3.37	26.52 ± 3.00	22.95 ± 2.54	22.30 ± 0.51
Co	19.55 ± 1.72	16.14 ± 1.64	14.38 ± 2.08	13.22 ± 1.24
Sn	10.24 ± 0.79	12.82 ± 0.59	13.58 ± 1.85	12.87 ± 1.22
S	33.64 ± 2.16	44.51 ± 0.43	49.06 ± 0.25	51.6 ± 0.37

**Table 4:** Compositional ratios of CCTS nanoparticles synthesized with 1.90 mmol Cu at 200 °C for different reaction times is obtained from EDS analysis

Cu concentration/elements	1 h	3 h	6 h	12 h
Cu/(Co+Sn)	1.22 ± 0.00	0.91 ± 0.03	0.82 ± 0.02	0.86 ± 0.06
Cu/Sn	3.56 ± 0.05	2.06 ± 0.13	1.69 ± 0.04	1.74 ± 0.12
Co/Sn	1.90 ± 0.02	1.25 ± 0.07	1.05 ± 0.03	1.02 ± 0.01
S/(Cu+Sn+Co)	0.50 ± 0.01	0.80 ± 0.06	0.97 ± 0.11	1.06 ± 0.06

**Table 5:** Chemical composition of CCTS nanoparticles synthesized with different Cu concentrations is obtained from XPS analysis.

Cu concentration/elements	2.5 mmol	2.25 mmol	2.00 mmol	1.90 mmol
Cu	27.15	26.5	24.89	23.6
Co	14.5	13.43	13.2	13.3
Sn	9.92	10.52	11.4	12.5
S	48.43	49.55	50.51	50.6

**Table 6:** Peaks positions and their FWHM values for constituent elements of CCTS nanoparticles synthesized with 2.50 mmol Cu (C1). ΔE (eV) denotes the difference between peak1 and peak2.

	Cu		Co		Sn		S	
	Position	FWHM	Position	FWHM	Position	FWHM	Position	FWHM
Peak1(eV)	931.748	1.354	778.770	1.175	486.056	1.105	161.232	0.793
Peak2(eV)	951.624	2.501	793.408	1.566	494.491	1.130	162.304	2.234
Peak3(eV)	946.660	4.062	779.113	4.283	487.010	1.310	164.361	1.687
Peak4(eV)			795.220	2.523	495.514	1.312		
ΔE (eV)	19.86		14.70		8.43		1.072

**Table 7:** Peaks positions and their FWHM values for constituent elements of CCTS nanoparticles synthesized with 2.25 mmol Cu (C2). ΔE (eV) denotes the difference between peak1 and peak2.

	Cu		Co		Sn		S	
	Position	FWHM	Position	FWHM	Position	FWHM	Position	FWHM
Peak1(eV)	931.763	1.426	778.451	1.341	486.142	1.096	161.297	0.888
Peak2(eV)	951.621	2.314	793.266	0.716	494.563	1.157	162.592	2.412
Peak3(eV)	946.687	4.103	780.273	4.120	487.051	1.614	164.694	1.158
Peak4(eV)			795.320	3.600	495.454	1.745		
ΔE (eV)	19.85		14.81		8.42		1.29

**Table 8:** Peaks positions and their FWHM values for constituent elements of CCTS nanoparticles synthesized with 2.00 mmol Cu (C3). ΔE (eV) denotes the difference between peak1 and peak2.

	Cu		Co		Sn		S	
	Position	FWHM	Position	FWHM	Position	FWHM	Position	FWHM
Peak1(eV)	931.575	1.719	778.350	1.111	485.956	1.062	161.425	0.866
Peak2(eV)	951.525	2.091	793.302	0.444	494.398	1.115	162.710	2.302
Peak3(eV)	947.317	4.694	779.761	4.158	486.561	1.760	164.712	1.406
Peak4(eV)			794.880	3.633	495.190	1.750		
ΔE (eV)	19.95		14.95		8.44		1.28

**Table 9:** Peaks positions and their FWHM values for constituent elements of CCTS nanoparticles synthesized with 1.90mmol Cu (C4). ΔE (eV) denotes the difference between peak1 and peak2.

	Cu		Co		Sn		S	
	Position	FWHM	Position	FWHM	Position	FWHM	Position	FWHM
**Peak1(eV)**	931.846	1.432	778.439	1.236	485.927	1.121	161.316	0.861
Peak2(eV)	951.772	2.321	793.279	0.682	494.301	1.017	162.585	2.207
Peak3(eV)	946.880	4.130	779.772	4.813	486.940	1.845	164.694	1.342
Peak4(eV)			795.300	5.876	495.045	1.530		
ΔE (eV)	19.92		14.84		8.37		1.26	

**Fig. 16:**
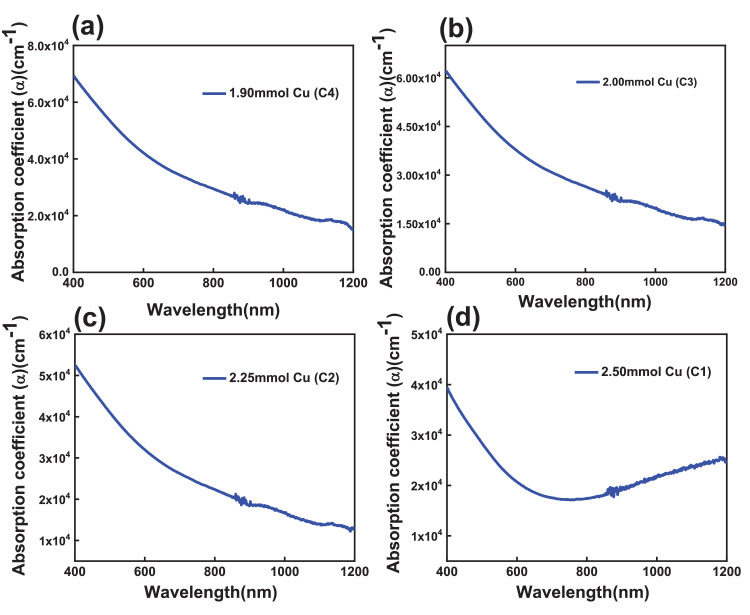
Absorption coefficient vs. wavelength plots of CCTS films fabricated with different Cu concentrations.

**Fig. 17:**
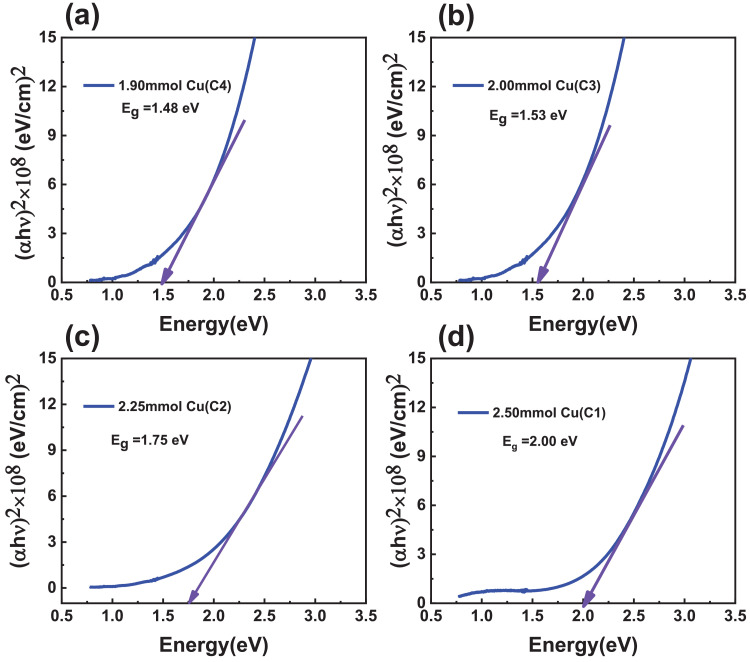
Tau's plots of CCTS films fabricated with different Cu concentrations.

The calculated absorption coefficient CCTS films fabricated with different Cu concentrations is found to be the order of 10^4^ cm^−1^ in visible and NIR region, and it is the same order of CZTS and CIGS chalcogenide materials (Fig. 16) [12]. Tau's plots of CCTS films fabricated with different Cu concentrations are shown in Fig. 17.

## TLM pattern process for two-terminal CCTS based devices

**Fig. 18:**
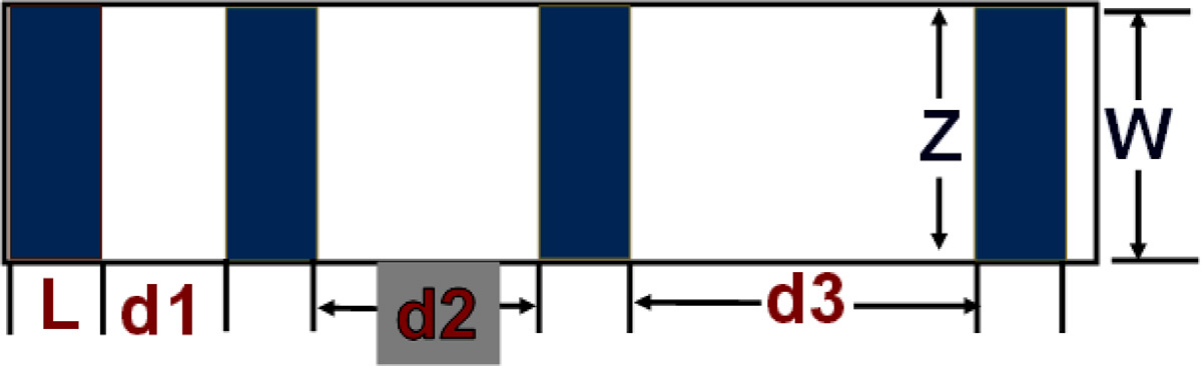
TLM patterns for the eValuation of CCTS film. L and Z is the width and length of metal pads, respectively. d1, d2, d3 correspond to a different separation gap for metal pads, 100 μm, 200 μm, 300 μm, respectively.

**Fig. 19:**
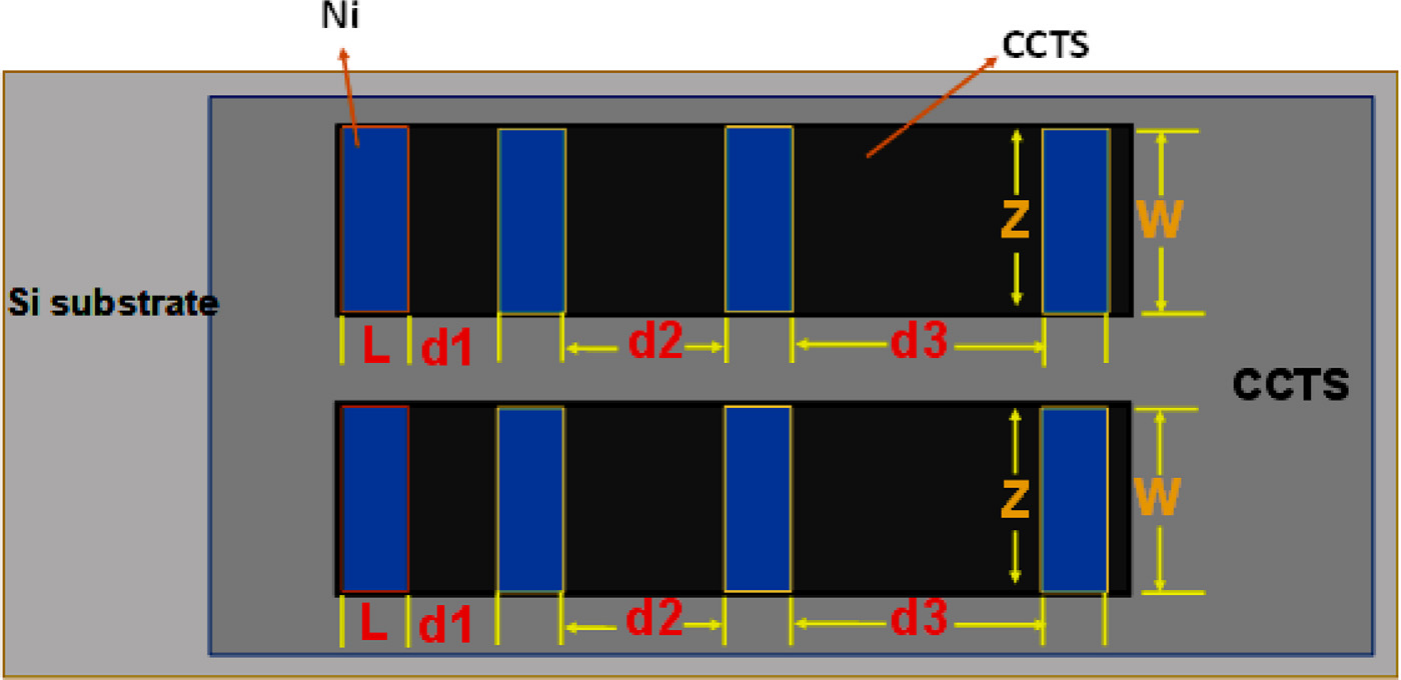
Schematic for two-terminal CCTS based device.

**Fig. 20:**
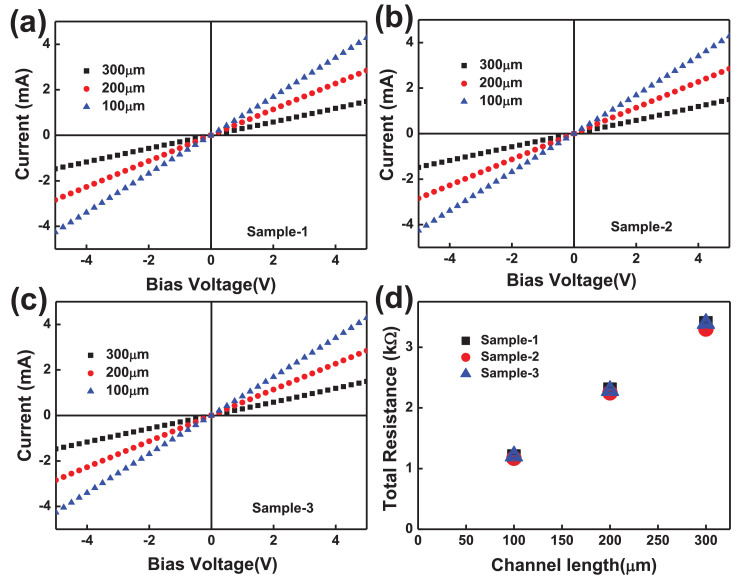
I-V characteristics and the total resistance of CCTS based two terminals fabricated with different channel length ranging from 100 μm to 300 μm, respectively. All CCTS nanoparticles were synthesized with 2.50 mmol Cu via MEA promoted hydrothermal process at 200 °C for 12 h.

**Fig. 21:**
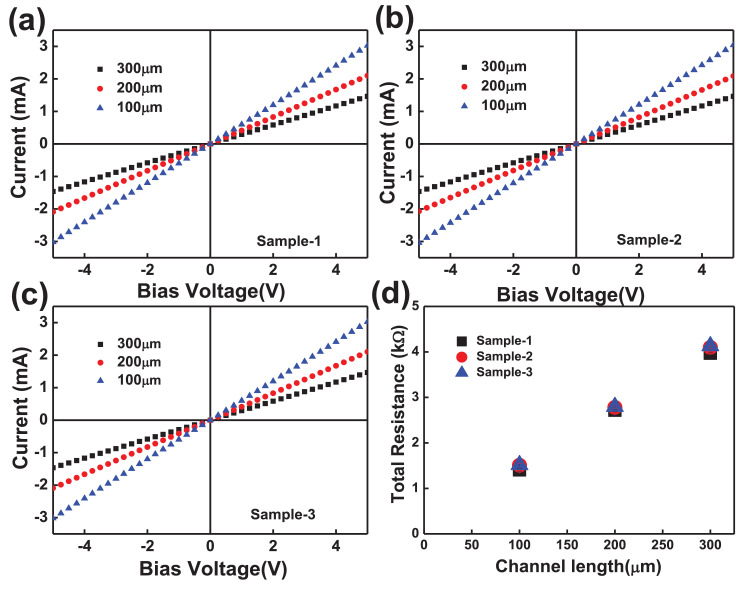
I-V characteristics and the total resistance of CCTS based two terminals fabricated with different channel length ranging from 100 μm to 300 μm, respectively. All CCTS nanoparticles were synthesized with 2.25 mmol Cu via MEA promoted hydrothermal process at 200 °C for 12 h.

**Fig. 22:**
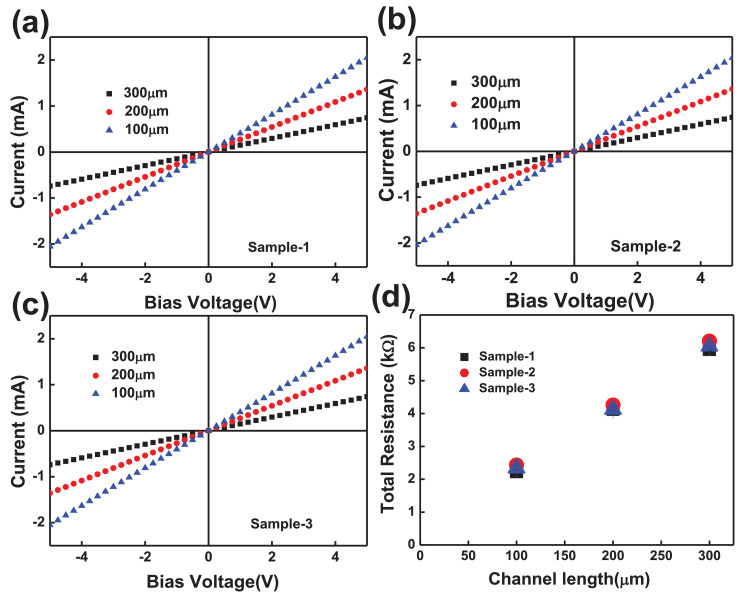
I-V characteristics and the total resistance of CCTS based two terminals fabricated with different channel length ranging from 100 μm to 300 μm, respectively. All CCTS nanoparticles were synthesized with 2.00 mmol Cu via MEA promoted hydrothermal process at 200 °C for 12 h.

**Fig. 23:**
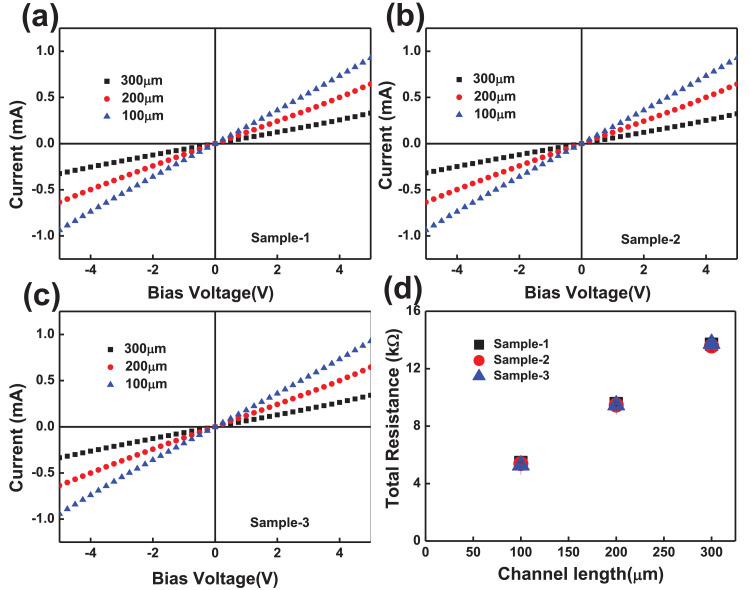
I-V characteristics and the total resistance of CCTS based two terminals fabricated with different channel length ranging from 100 μm to 300 μm, respectively. All CCTS nanoparticles were synthesized with 1.90 mmol Cu via MEA promoted hydrothermal process at 200 °C for 12 h.

The electrical properties of two-terminal CCTS devices, based on CCTS films annealed in different conditions, were estimated by the TLM pattern method. The TLM pattern is shown in Fig. 18. Two-terminal devices were fabricated by thermal eVaporation of Ni (∼50 nm, DAEKI HI-TECH CO, Ltd) as a drain and source electrode on the annealed CCTS films. With a fixed channel width (w) of 800 μm, two-terminal devices, with different channel length (d_ch_) ranging from 100 μm to 300 μm, were defined by thermal eVaporation via a metal shadow mask. The TLM patterns for CCTS film are illustrated in Figs. 18, and 19. The total resistance of devices is estimated by the inverse slope of the I-V characteristics (Fig. 20, Fig. 21, Fig. 22, Fig. 23). The sheet resistance of CCTS (channel materials) = slope (the slope is determined from the R vs. l plot) /width of the channel (800 μm) (Figs. 20d, 21d, 22d, and 23d). The contact resistance (2R_c_) of the devices is extracted from y-intersect Figs. 20d, 21d, 22d, and 23d. The calculated contact resistance (R_c_) and sheet resistance (R_sh_) are given in Table 10.

**Table 10:** Electrical properties for two-terminal devices based on CCTS nanoparticles.

Cu concentration	Cu/(Co+Sn)	Contact Resistance (Ω) (R_c_)	Sheet Resistance R_sh_ (kΩ/sq)
2.5 mmol	1.1728	108.31 ± 3.43	13.56 ± 0.19
2.25 mmol	1.08792	247.25 ± 7.47	16.79 ± 0.51
2.00 mmol	0.94662	737.64 ± 44.81	23.43 ± 0.22
1.90 mmol	0.86054	1116.18 ± 61.73	40.7 ± 0.26

## Experimental design, materials and methods

### Experimental details

In this study, earth-abundant quaternary CCTS nanoparticles were synthesized by monoethanolamine (MEA) promoted the hydrothermal process, and their physical properties are systematically analyzed, corresponding to the variation of Cu concentration and reaction times. 2.50 mmol Cu(OOCCH_3_)_2_•H_2_O (Alfa Aesar), 1.40 mmol CoCl_2_•6H_2_O, (ACS, 98.0-102.0%, Alfa Aesar), 1.25 mmol SnCl_2_ (*Sigma–Aldrich*), and 12 mmol CH_4_N_2_S (*Sigma–Aldrich*) initial precursors were added one by one to 63 ml deionized water (DI water) in a Teflon liner. The Teflon liner was kept at 50 °C for 30 min under magnetic stirring to get a homogenous solution. After that, 7 ml MEA stabilizer/complexing agent after 15 min was added to the homogenous solution drop by drop to increase the pH value of the prepared solution. This promotes control of cations and anions in the solution. The solution was further stirred for 10 min at the same temperature, and subsequently, Teflon liner was transferred into stainless-steel autoclave. The lid of the autoclave was appropriately closed, and the hydrothermal reaction was carried out at 200 °C for 12 h. After the hydrothermal reaction is completed, the system was programmed to cool down to room temperature naturally. The precipitate was collected from Teflon liner and washed several times with DI water/ isopropyl alcohol (IPA) (7:3) to remove the by-products and contaminants which might contain in the precipitates. The final precipitate was dried under vacuum at 80 °C for 6 h to get CCTS nanoparticles. The same synthesis protocol was used to study the effects of variation in Cu concentration, such as a 2.25 mmol, 2.00 mmol, and 1.90 mmol on structural, morphology CCTS nanoparticles while keeping all other concentrations of the constituent precursor's remains constant.

The synthesized nanoparticles were subjected to physical characteristics such as phase analysis (XRD and Raman spectroscopy), morphology (FEG-SEM and FEG-TEM), chemical composition (energy dispersive spectroscopy), valence states and chemical composition of synthesized nanoparticles by X-ray photoelectron spectroscopy analysis. All synthesized CCTS nanoparticles were further utilized to study the effects of Cu concentrations on electrical and photoresponse of CCTS nanoparticles-based films. 200 mg synthesized CCTS nanoparticles dispersed in 1 ml N-Methyl-2-pyrrolidone (NMP) in a vial and ultrasonicated for 15 min to form a homogeneous paste. The prepared paste was coated on thermally oxidized Si wafer (300 nm) by drop-casting technique to form a film. The drop casted films were annealed in vacuum at 200 °C for 1 h. Two terminal CCTS devices were fabricated by depositing Ni as a drain and source via thermal eVaporation. The detailed two-terminal device fabrication steps (Fig. S2). The electrical properties, such as contact resistance and sheet resistance of fabricated devices, were measured via the transfer length method (TLM). The photoresponse of the fabricated device was performed under the illumination of a halogen lamp (2.5 mW/cm^2^).

Characterization tools: X-ray diffraction (XRD, Rigaku, smart Lab) and Raman Spectroscopy (JOBIN YVON, Lab RAM Hr 800) have been used to examine the phase formation of the synthesized nanoparticles. XRD was carried in the 2-theta scan range of 20ᵒ–80ᵒ using Cu _Kα_ radiation (λ = 1.54 Å) irradiation. Measurement of Raman spectroscopy was performed in the range of 200–500 cm^−1^ using an argon excitation wavelength of 632.85 nm. X-ray photoelectron spectroscopy (XPS, PHI 5000 Versa Probe Ⅱ) determined the value for the valence state of constituent elements and the chemical composition of prepared CCTS samples. Surface morphology for the prepared CCTS nanoparticles was carried out by using FEG-SEM (JEOL, EDS-7800F). Energy dispersive spectroscopy (EDS, JEOL, EDS-7800F) measurements of the synthesized nanoparticle were performed to quantify the chemical composition. The chemical composition for the synthesized CCTS nanoparticles is measured by using EDS with operating voltage (15 kV) and working distance (10mm). The CCTS paste/ink drop casted on a soda-lime glass substrate (SLG substrate) to form films and dried at 200 °C for 1 h in a vacuum oven with the base pressure of ∼ 15 mtorr to obtain the films. The absorbance properties of prepared films were measured using UV–Vis–NIR Spectrometer (Agilent Technologies) in the wavelength ranging from 400 nm to 1100 nm. I-V measurements for two-terminal devices on CCTS films were carried out by using a semiconductor parameter analyzer (Agilent, 4155B). The photoresponse study was conducted under the illumination of a halogen lamp (2.5 mW/cm^2^).

## Ethics Statement

Authors declare that the article is original and unpublished and is not being considered for publication elsewhere, and also it has not been submitted simultaneously anywhere. All authors have checked the revised manuscript and have agreed to the submission. The manuscript has been prepared according to the “Author Guidelines.”

## Declaration of Competing Interest

The authors declare that they have no known competing financial interests or personal relationships that could have appeared to influence the work reported in this paper.

## Appendix Supplementary materials

Image, application 1
